# Statistical optimization of a podoviral anti-MRSA phage CCASU-L10 generated from an under sampled repository: Chicken rinse

**DOI:** 10.3389/fcimb.2023.1149848

**Published:** 2023-03-31

**Authors:** Israa M. Abd-Allah, Ghadir S. El-Housseiny, Mohamed H. Al-Agamy, Hesham H. Radwan, Khaled M. Aboshanab, Nadia A. Hassouna

**Affiliations:** ^1^ Department of Microbiology and Immunology, Faculty of Pharmacy, Ain Shams University, Organization of African Unity Street, Abbassia, Cairo, Egypt; ^2^ Department of Pharmaceutics, College of Pharmacy, King Saud University, Riyadh, Saudi Arabia; ^3^ Department of Microbiology and Immunology, Faculty of Pharmacy, Al-Azhar University, Cairo, Egypt

**Keywords:** phage, MRSA, optimization, RSM, podoviral phage

## Abstract

**Introduction:**

The insurgence of antimicrobial resistance is an imminent health danger globally. A wide range of challenging diseases are attributed to methicillin-resistant *Staphylococcus aureus* (MRSA) as it is weaponized with a unique array of virulence factors, and most importantly, the resistance it develops to most of the antibiotics used clinically. On that account, the present study targeted the optimization of the production of a bacteriophage active against MRSA, and evaluating some of its characters.

**Methods and results:**

The bacteriophage originated from a quite peculiar environmental source, raw chicken rinse and was suggested to belong to *Podoviridae*, order *Caudovirales*. It withstood a variety of extreme conditions and yield optimization was accomplished *via* the D-optimal design by response surface methodology (RSM). A reduced quadratic model was generated, and the ideal production conditions recommended were pH 8, glycerol 0.9% v/v, peptone 0.08% w/v, and 10^7^ CFU/ml as the host inoculum size. These conditions led to a two-log fold increase in the phage titer (1.17x10¹² PFU/ml), as compared to the regular conditions.

**Discussion:**

To conclude, statistical optimization successfully enhanced the output of the podoviral phage titer by two-log fold and therefore, can be regarded as a potential scale-up strategy. The produced phage was able to tolerate extreme environmental condition making it suitable for topical pharmaceutical preparations. Further preclinical and clinical studies are required to ensure its suitability for use in human.

## 1 Introduction

Right from the start, bacteriophage: the most ubiquitous entity on Earth has been combatting its natural opponent, bacteria (Hatfull, 2015; Fitzgerald et al., 2021). In the meantime, bacteria have been undergoing their own battle against humans. However, for almost a century now, the miraculous molecules, antibiotics, have made it possible to overcome certain fatal bacterial infections that were once incurable. Unluckily, over the last decades, the misuse and overuse of antibiotics on top of multiple other factors have contributed to the emergence of a high-tier global threat, antimicrobial resistance (AMR) (Mancuso et al., 2021). In addition, the stunning success of both the pharmaceutical research and industry in developing a plethora of effective antibiotics has created complacency facing a crisis as threatening as antimicrobial resistance (Spellberg and Gilbert, 2014). At present, an estimated number of 700,000 people die every year, all over the world, as a very sad consequence to AMR (Ang et al., 2004; Levy and Marshall, 2004; Mancuso et al., 2021). The World Health Organization (WHO) forewarns that the future could be further doomed if no innovative approaches come to reality and encounter such a problem. For example, it predicts that the number of yearly deaths caused by AMR can reach 10 million by year 2050 (WHO, 2017; Friedman, 2020; Mancuso et al., 2021). Along the same lines, the expeditious transformation of well-known organisms like *Staphylococcus aureus* into superbugs is raising a red flag. Prior to 1959, diseases caused by *Staphylococcus aureus* were deftly treated using penicillin. Then methicillin was introduced as the first-in-class beta-lactamase resistant agent. Quickly, and unfortunately, methicillin - resistant *Staphylococcus aureus* (MRSA) arose in community settings as well as hospitals (Enright et al., 2002; Ang et al., 2004; Grema, 2015). MRSA is disreputable for the kind of diseases it causes. Infections as hard as diabetic foot, joint, skin and soft tissue infections and pneumonia, along with their hard-to-treat complications are commonly caused by MSRA (Grema, 2015; Craft et al., 2019; MRSA | CDC, 2019; Algammal et al., 2020; Siddiqui and Koirala, 2020). Recently, MRSA intestinal colonozation was reported, which may contribute to the horizontal gene transfer of resistant genes to innocuous organisms (Nataraj and Mallappa, 2021). Again, the WHO, in 2017, declared MRSA a high priority multi-drug resistant (MDR) organism (WHO, 2017). Furthermore, according to The Infectious Diseases Society of America (IDSA) declaration, *S. aureus* is one of the noxious “ESKAPE” microorganisms, implying the capability to elude even the recourse antibiotics like vancomycin (Santajit and Indrawattana, 2016). This is a serious call for a pressing, coordinated countermove if we are to avoid a scenario where we could be sent back to the dark ages of medicine (Meek et al., 2015; Dodds, 2017).

The veteran fighters of bacteria, bacteriophages, have been lingering for a century now. Bacteriophages are renowned as the viral dark matter; a virus that subsists only on bacteria and there are more of them on the planet than any other organism (Hatfull, 2015; Fitzgerald et al., 2021). There are two major classes of them: lytic and lysogenic. The lytic phage is the type capable of destroying its bacterial host (Ackermann, 2012b). The first successful therapeutic use of bacteriophages were reported early in 1926 (D’Herelle, 1929). Later on, some pharmaceutical companies such as Eli Lilly and Abbott attempted using phages against staphylococcal infections and proved promising (Ackermann, 2012b). Over the past few decades, the interest in phage therapy has grown again, and some successful leaps – though sporadic- have been accomplished (Luong et al., 2020). In view of the foregoing, the present study aimed at optimizing the production of a bacteriophage showing lytic activity against clinical MRSA, as an example of a perturbing multidrug resistant bacterium, believing in phage aptitude in tipping the scales to the humanity’s side in their fight against superbugs (Berryhill et al., 2021; Gordillo Altamirano and Barr, 2021). Moreover, this study tried to evaluate the overall stability of the propitious phage.

## 2 Materials and methods

### 2.1 MRSA isolates and MRSA specific phage L10

The bacterial hosts used along the course of phage isolation and further testing were twenty-three MRSA isolates collected, identified and susceptibility profiled as mentioned in our previous study performed in our lab (Abd-Allah et al., 2022). The phage lysate used in this study, phage L10, was obtained from chicken rinse. Briefly, 5 mls of the fresh rinse of a raw chicken have been incubated with 5 ml of the bacterial host and 50 mls of double strength TSB. The mixture was incubated overnight at 200 rpm centrifuged at 5000 rpm for 20 min and the supernatant was treated by shaking with chloroform to eradicate the bacterial cell remnants. This lysate was kept at 4°C. The lysate was screened for activity against MRSA by spot test as mentioned in our previous study (Abd-Allah et al., 2022). The recovered anti MRSA phage was put in the culture collection of Ain Shams University (CCASU) belonging to the World Data Centre for Microorganisms (WDCM) under the accession code Phage CCASU-L10. (https://ccinfo.wdcm.org/details?regnum=1186).

### 2.2 Plaque assay, and phage purification

Plaque assay was conducted on the CCASU-L10 lysate using a technique named standard double agar overlay (DAO) technique to determine the concentration of the phage particles in the original lysate (Anderson et al., 2011; Vahedi et al., 2018). Briefly, bacterial host used for phage isolation was freshly prepared and the count was adjusted by dilution in sterile trypticase soy broth (TSB) and calibrated to 0.5 McFarland suspension for a concentration equivalent to 10^8^ CFU/ml.

Saline-magnesium (SM) buffer was used for the plaque assay dilutions. It was prepared by dissolving 5.8 g NaCl and 2 gm MgSO4•7H2O into 50 ml tris-chloride (5 mM) (Biodiagnostics ^®^, Cairo, Egypt). The solution was then diluted with distilled water to 1000 ml and adjusted to pH 7.5 using NaOH and HCl, then sterilized (Bonilla et al., 2016). A volume of 100 μl of the phage lysate was ten-fold serially diluted, in SM buffer (900 μl) and the dilutions were mixed afterwards with a constant volume of the bacterial host. This was mixed with 3 ml soft overlay (double strength TSB containing 0.75 g/100 ml agar). The mixtures were then poured and evenly distributed over previously prepared plates of TSA underlay. Plates were left undisturbed for the overlay to completely solidify and were incubated overnight at 28°C in upright position. Plaques were then inspected and totaled. To calculate the phage titer, the following formula was used: Phage titer in plaque-forming unit per ml (PFU/ml) = Number of plaques/(Volume of lysate infected * Dilution) (Clokie and Kropinski, 2009):

Single plaque isolation was undertaken three consecutive times to ensure the lysate purity. A single well-defined plaque was picked out using a sterile spatula, suspended in SM buffer, and left for 2 hours allowing the phages to get released. Next, the suspension was incubated with the fresh bacterial host, overnight, shaking at 180 rpm. After that, the cocultured suspension was subjected to centrifugation, and was treated with chloroform to rid the bacterial cells. The final suspension was kept at 4°C (Cross et al., 2015).

### 2.3 Main properties of the phage CCASU-L10

#### 2.3.1 Host range

The host range of CCASU-L10 lysate was assessed by spot testing against 22 isolates of MRSA (Mahmoud et al., 2020). In short, previously-prepared TSA underlay plates were overlaid with 3-ml aliquots of double-strength soft TSB agar, each inoculated with the MRSA isolate in-question. After solidification, each plate was spotted with 15 microliters of the phage suspension, incubated overnight and examined for the next day. Presence of a lytic effect was detected through inhibition of the bacterial host growth in the place of the spot applied in the middle of an otherwise well-grown, confluent sheet.

#### 2.3.2 Phage particles' morphology

To get the phage CCASU-L10 lysate ready for microscopical examination, a high-titer suspension was purified from any high-density, interfering matter by centrifugation (25 min at 10,000 rpm) twice, and syringe filtration (0.22 μm). A 20-μl specimen was then prepared in accordance with the procedure described by Kalatzis et al., and was examined using transmission electron microscope (JEOL_JEM_1010 Electron Microscope Siemens & Halske, Germany, done at Regional Center for Mycology and Biotechnology, Al-Azhar University, Cairo, Egypt) (Kalatzis et al., 2016; Mahmoud et al., 2020).

#### 2.3.3 Longevity test

Bacteriophage lysate aliquots were maintained at: 4, 37, and -20°C for 90 days. They were inspected for lytic activity at days 1, 2, 3, 4, 5, 6, 7, 15, 30, 60, and 90 using spot test (Fortier and Moineau, 2009; Mahmoud et al., 2020).

#### 2.3.4 Thermal stability test

An aliquot of the lysate was subjected to the temperature range 30 to 60°C at 5-degree intervals, each for 1 hr. Then, a fixed volume was drawn out and was promptly tested *via* spot test. After overnight incubation, the plates were examined for loss of lytic activity and accordingly thermal inactivation point was revealed (Mahmoud et al., 2020).

#### 2.3.5 pH stability test

1N NaOH and HCl were used to adjust the pH of TSB (1 to 13), after that it was mixed with a similar volume of the phage lysate. Suspensions were left for an hour at room temperature. The phage infectivity was checked out at the different pH values by spot test (Jamalludeen et al., 2007; Mahmoud et al., 2020).

#### 2.3.6 Stability towards UV light

An aliquot of the phage lysate was exposed to the UV light in a laminar flow hood at a distance of 30-cm from the UV source for 1 hr. At 10, 20, 30, 40, 50 and 60 minutes, a sample was drawn and immediately spot tested (Rodriguez et al., 2014; Alič et al., 2017; Kim et al., 2018).

#### 2.3.7 Sensitivity to organic solvents

The concentrations (10, 30, 50 and 100% v/v) of ethanol, isopropyl alcohol, and chloroform were prepared by dilution into distilled water. Phage lysate was mixed with each of these concentrations (1:1), and was kept at room temperature for 1 hr before spot testing (Kim et al., 2018; Łubowska et al., 2019).

### 2.4 Phage CCASU-L10 production optimization

#### 2.4.1 The effect of individual factors using one - factor - at - a time

How the coculture functions under the influence of several factors was examined; one factor at a time. The phage titer was determined (PFU/ml) at the end of every run by performing plaque assay. The factors examined were: carbon source (0.5% v/v glycerol, and 0.5% w/v sucrose), nitrogen source (0.1% w/v peptone, and 0.1% w/v glycine) (Kim et al., 2021), inoculum size of the bacterial host (10^7,^ 10^8,^ 10^9^ CFU/ml), and temperature (28, 33, 37°C) (Grieco et al., 2012; González-Menéndez et al., 2018a; Reuter and Kruger, 2020). Factors that resulted in the best phage production were chosen for the next experiment.

#### 2.4.2 RSM Optimization

RSM relates a response to the levels of the variables that influence it. The factors selected in this study were: pH (coded A), glycerol concentration (coded B), and peptone concentration (coded C). As shown in the **Table 1**, each variable was studied at 3 levels. The D-optimal design, in the statistical software package; Design Expert^®^ v. 7.0 (Design Expert Software, Stat-Ease Inc., Statistics Made Easy, Minneapolis, MN, USA), was selected as the appropriate design to optimize our response. After inputting our factors into the design, 17 experiments were suggested by the software. The seventeen production runs were conducted in the lab (**Table 2**), and one response value, phage titer in PFU/ml, was recorded at the end. The factors’ interaction was studied, and the software configured a polynomial reduced quadratic equation using the data gathered from the experiments (Bhaumik et al., 2013; González-Menéndez et al., 2018a; El-Housseiny et al., 2021).

**Table 1:** The factors selected for RSM optimization of phage production and their levels; A: pH, B: carbon source, C: nitrogen source.

Factor		-1	0	+1
A: pH		6	7	8
Media composition	B: Carbon source concentration Glycerol concentration (%v/v)	0.1	0.5	0.9
	C: Nitrogen source concentration Peptone concentration (%w/v)	0.05	0.1	0.15

**Table 2:** The observed responses for the D-optimal design experiments suggested for phage CCASU-L10.

Run	A: pH	B: Glycerol (% v/v)	C: Peptone (% w/v)	Response (Phage Count) (PFU/ml)
1	**6**	**0.10**	**0.05**	**2x10¹°**
2	**6**	**0.9**	**0.15**	**3x10¹¹**
3	**7**	**0.10**	**0.10**	**1x10¹¹**
4	**8**	**0.9**	**0.15**	**2.8x10¹¹**
5	**7.5**	**0.7**	**0.10**	**9.7x10¹¹**
6	**6**	**0.10**	**0.15**	**5.4x10¹¹**
7	**6**	**0.10**	**0.15**	**5.7x10¹¹**
8	**6**	**0.10**	**0.05**	**2.2x10¹°**
9	**8**	**0.5**	**0.05**	**4.6x10¹¹**
10	**8**	**0.10**	**0.15**	**1.4x10¹¹**
11	**7**	**0.90**	**0.05**	**4.3x10¹¹**
12	**6**	**0.90**	**0.05**	**1.8x10¹¹**
13	**7**	**0.50**	**0.10**	**8.4x10¹¹**
14	**8**	**0.90**	**0.15**	**3x10¹¹**
15	**7**	**0.50**	**0.15**	**7.3x10¹°**
16	**8**	**0.10**	**0.05**	**9x10¹°**
17	**6**	**0.50**	**0.10**	**8x10¹¹**

#### 2.4.3 Statistical analysis

Two replicates were performed for each run, and the average recorded. Data analysis was carried out by Design Expert^®^ v. 7.0. The same program generated response surfaces and model diagnostic plots as well (El-Sayed et al., 2020a).

#### 2.4.4 RSM results verification

In the Design Expert^®^ Software, the numerical optimization function was deployed to discern the optimum conditions as commended by the program. A new experiment was carried out under the recommended optimal conditions (pH 8, glycerol 0.9 % v/v, and peptone 0.08 % w/v). The actual phage titer produced was compared to that produced by the control run performed under the basic conditions.

## 3 Results

### 3.1 Plaque assay 

**Figure 1:**
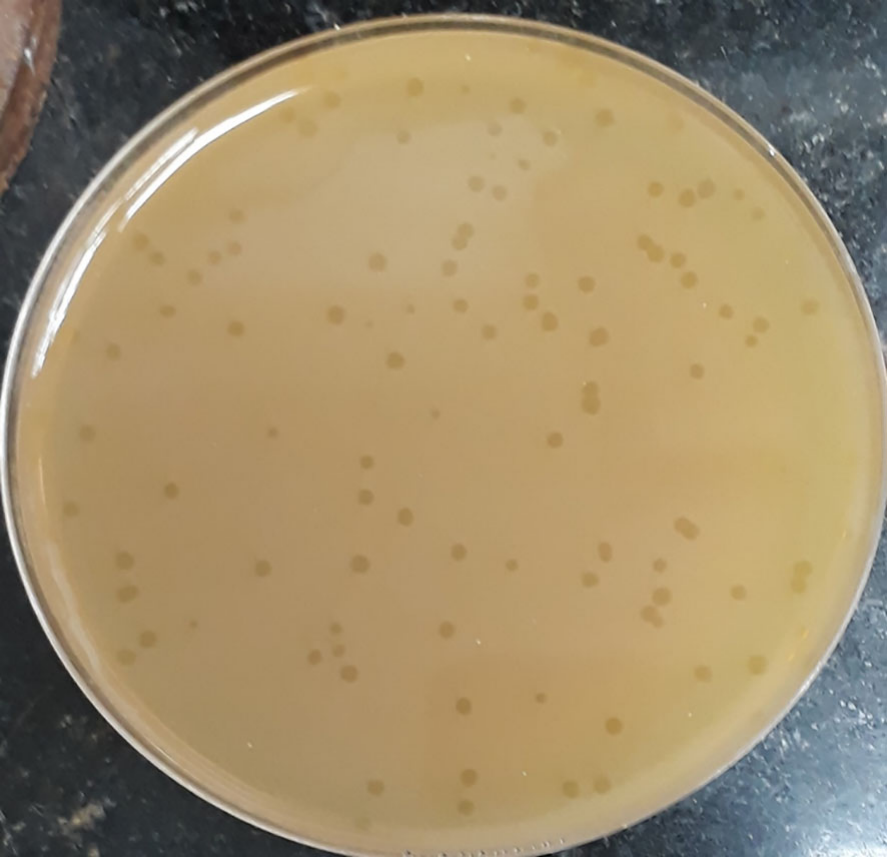
Plaque morphology of phage CCASU-L10. Plaques are clear, circular, and small in size (diameter of 2-5 mm).

The initial phage titer was 1.4x10^8^ PFU/ml. The plaques appeared small in size (2-5 mm), circular and clear (**Figure 1**).

### 3.2 Evaluation of the CCASU-L10 phage properties

#### 3.2.1 Host range

Phage CCASU-L10 showed clear lytic spot against all the in-test 22 MRSA isolates.

#### 3.2.2 Phage CCASU-L10 morphology by TEM

**Figure 2:**
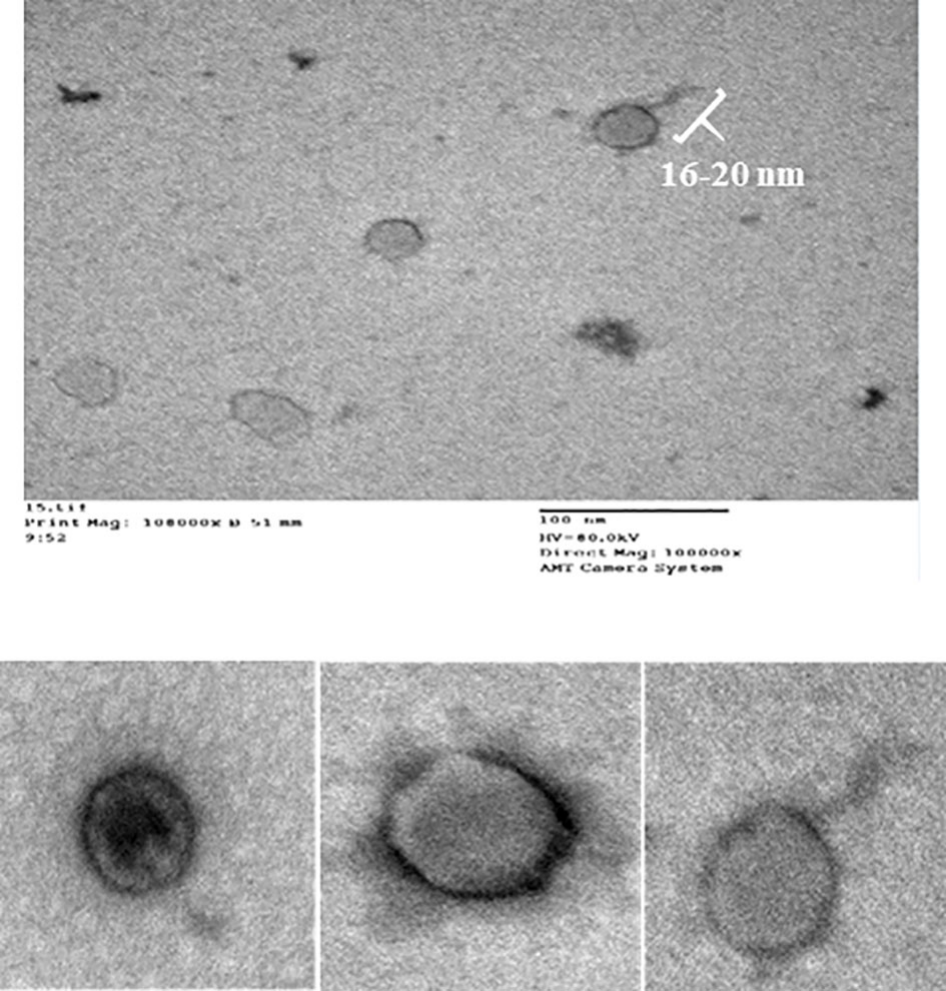
Electron micrograph of phage CCASU-L10, showing a 16-nm long tail, and a head diameter of 73 nm.

CCASU-L10 appeared to be a tailed bacteriophage as shown in the electron micrograph (**Figure 2**), so it is suggested to be classified under the order *Caudovirales*. The tail length was roughly estimated to be 16 nm, and the head diameter was 73 nm. It was difficult to accurately define the exact tail dimensions, this is because intact tails were scanty in the examination field, also the size greatly depended on the phage particle position on the grid. The tail lacked both head-to-tail connectors, and base plates. These findings corresponded to the description present on “Viral Zone” website (Podoviridae ~ ViralZone, 2011; King et al., 2012; Podoviridae, 2012; Alič et al., 2017), and the rules compiled by the International Committee on Taxonomy of Viruses (ICTV) (King et al., 2011; King et al., 2012). Based on this, CCASU-L10 probably belongs to the morphotype: *Podoviridae*.

#### 3.2.3 Longevity test

The CCASU-L10 aliquots survived at the different storage temperatures and maintained infectivity (as manifested by the positive spot test result) until 90 days.

#### 3.2.4 Thermal stability

According to the spot test, phage CCASU-L10 kept its activity at all the temperatures tested except 60 °C, at which the lytic spot totally vanished. Therefore, thermal inactivation point was 60 °C.

#### 3.2.5 pH stability

Phage CCASU-L10 managed to produce clear lytic spots along the pH range from 3-11. However, when challenged by either extremely acidic (pH= 1, 2), or extremely alkaline (pH= 12, 13) medium, the spot vanished.

#### 3.2.6 Sensitivity to UV

CCASU-L10 retained its lytic activity up till 30 minutes of direct UV irradiation. However, it waned progressively after this point in time till it disappeared after 1 hr of exposure.

#### 3.2.7 Sensitivity to organic solvents

Interestingly, CCASU-L10 held out against all the three chemical agents except that it got deactivated by 100% v/v isopropyl alcohol.

### 3.3 Optimization of phage CCASU-L10 yield

#### 3.3.1 OFAT optimization

**Figure 3:**
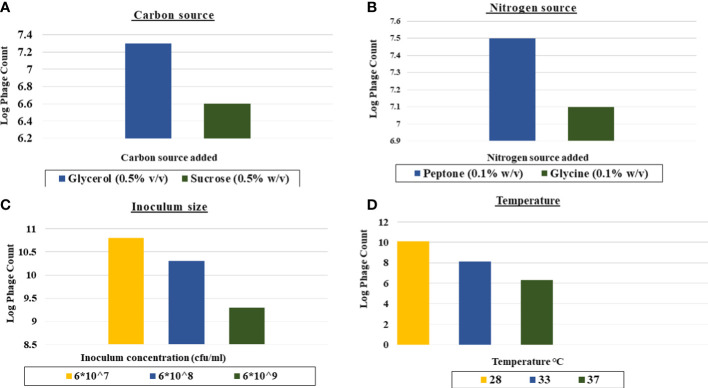
Effect of different factors on the phage CCASU-L10 production using one-factor-at-a time (OFAT). **(A)** Carbon source, **(B)** Nitrogen source, **(C)** Temperature, **(D)** Inoculum size).

As shown in **Figure 3**, the favorable carbon source for production was glycerol at 0.5% v/v as it led to 1 log increase in the phage yield compared to that of the control (**Figure 3A**).The yield of CCASU-L10 was also enhanced, almost 2-log fold by the nitrogen source peptone at 0.1% w/v (**Figure 3B**).Production runs performed at three different temperatures revealed that 28°C was the optimum incubation temperature to produce this phage, leading to phage titers reaching ~10^10^ PFU/mL (**Figure 3C**). Finally, the optimum host inoculum size was 6×10^7^ CFU/ml. (**Figure 3D**).

#### 3.3.2 RSM optimization

##### 3.3.2.1 The combined effect of pH, carbon and nitrogen sources

The seventeen experiments outlined by the Design Expert^®^ Software together with their observed results are depicted in **Table 2**. The reduced polynomial quadratic model fitted for the phage count prediction (PFU/ml) was:

Ln (phage count) = 58.67741-13.23649*A-0.85917* B + 253.83977* C + 0.96932 *AB -14.51184* AC-38.20151*BC+1.03978* A2-650.12098 * C2.

#### 3.3.3 Statistical analysis

**Figure 4:**
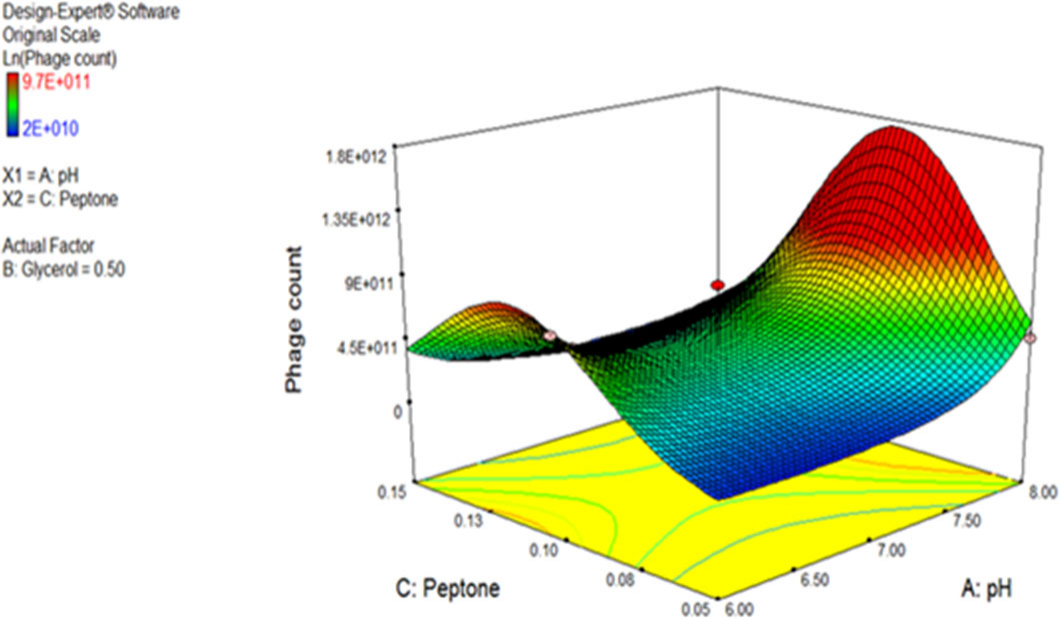
Three-dimensional (3D) response surface plot presenting the effect of three factors on CCASU-L10 production. When the effect of two parameters was plotted, the third one was set at central level.

ANOVA was performed to estimate the model’s significance and its suitability for titer prediction (**Table 3**). F-value and P-value were computed to express the model and terms’ significance. The F-value of the model was 18.76 (P-value = 0.0002), conferring the model significance. With respect to the model parameters, the linear terms: A, and B, alongside with the interactive ones: AB, AC, BC, A^2^, and C^2^ all had P-values less than 0.05, therefore proved significant (El-Sayed et al., 2020a; El-Sayed et al., 2020b). The coefficient of variation (C.V.%) was 1.45 showing that the experimental data are reliable (El-Sayed et al., 2020a; El-Sayed et al., 2020b; El-Housseiny et al., 2021). The ability of the model to dependably describe the data was estimated by R2, the coefficient of determination, which was 0.9494, proving the model ability to explain 94% of the variability in response (El-Sayed et al., 2020a; El-Housseiny et al., 2021). Predicted R-squared (Pred R^2^) of the model, 0.7766, was plausibly conforming to its adjusted counterpart, the adjusted R-squared (Adj R^2^) of 0.8988. Adequate precision ratio was 14.536, way above 4, favoring the use of this model to navigate the design space (El-Sayed et al., 2020a; El-Housseiny et al., 2021). The optimum conditions proposed by the generated 3D plots and numerical optimization function in the Design Expert® software for highest phage production were pH 8, glycerol 0.9% v/v, and peptone 0.08% w/v. **Figure 4** displays the 3D response surface plot generated by Design Expert^®^ for the model of CCASU-L10, it graphically demonstrates how the influential variables interconnect (El-Sayed et al., 2020a). Model diagnostics are used to investigate the model’s assumptions and evaluate its validity. Model CCASU-L10 diagnostics were illustrated as graphical summaries as follows:

**Table 3:** ANOVA for response surface reduced quadratic model (CCASU-L10).

Source	Sum of Squares	Degree of freedom(df)	Mean Square	F-value	P-Value	
**Model**	21.41	8	2.68	18.76	0.0002	Significant
**A-pH**	1.29	1	1.29	9.06	0.0168	
**B-Glycerol**	7.40	1	7.40	51.85	< 0.0001	
**C-Peptone**	0.28	1	0.28	1.99	0.1965	
**AB**	1.19	1	1.19	8.36	0.0201	
**AC**	5.07	1	5.07	35.51	0.0003	
**BC**	5.17	1	5.17	36.24	0.0003	
**A^2^**	2.22	1	2.22	15.55	0.0043	
**C^2^**	5.67	1	5.67	39.75	0.0002	
**Residual**	1.14	8	0.14			
**Lack of Fit**	1.13	5	0.23			
**Pure Error**	8.384E-003	3	2.795E-003			
**Cor Total**	22.55	16				

##### 3.3.3.1 The box cox plot

**Figure 5:**
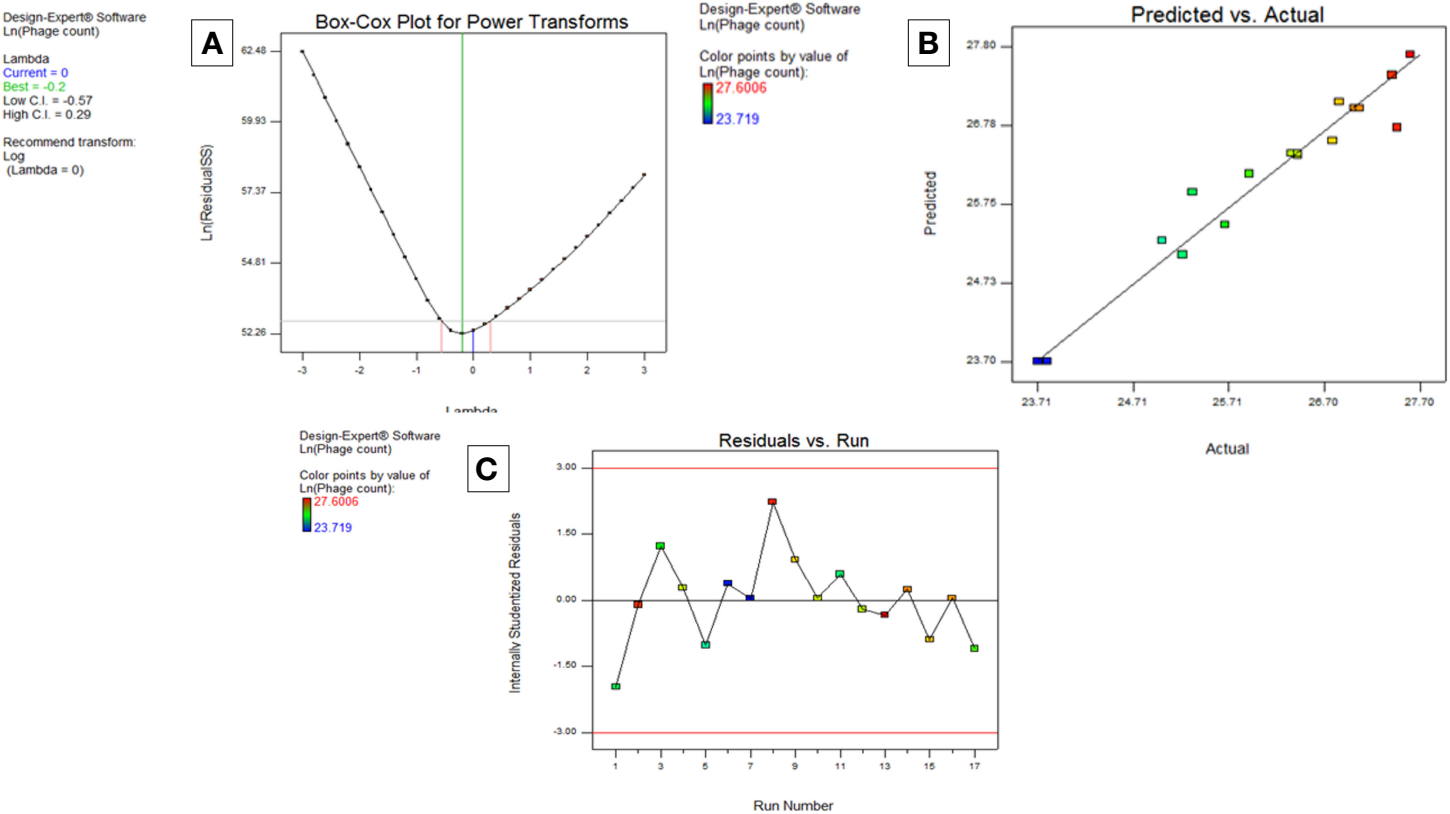
Model diagnostic plots for Model CCASU-L10. **(A)** Box Cox plot, **(B)** Actual vs. predicted plot, **(C)** Residuals versus run order plot.

The purpose of this graph is to decide on the felicitous power transformation to be performed. A transformation of lambda value from 1 to the natural log was recommended (**Figure 5A**).

##### 3.3.3.2 Predicted vs. actual plot

This plot expresses the degree of matching between the actual and the predicted data. Ideally, the actual data is identical to the predicted data, the case represented through the central line. For the model CCASU-L10, the predicted and actual responses are closely distributed around the line, thus they were fairly conforming (**Figure 5B**).

##### 3.3.3.3 Residuals vs. run order plot

This is a scatter plot where the y axis presents the residuals while the x axis presents the run order. The model CCASU-L10 led to a complying plot; the residuals bounced in no particular pattern around the residual = 0 line, evincing validity of the model (**Figure 5C**).

#### 3.3.4 Experimental model verification

An additional experiment of the phage CCASU-L10 propagation was carried out. This time, it was conducted with the suggested optimum levels for the 3 factors (pH 8, glycerol 0.9% v/v, and peptone 0.08% w/v) in order to verify the model’s competence and validity. The titer of the control run performed under the unoptimized conditions was 1.7x10¹° PFU/ml, whereas the optimized conditions resulted in a titer of 1.17x10¹² PFU/ml. Hence, the model suggestions capably led to a 2-log fold rise in phage yield.

## 4 Discussion

Resistance to most of the antibiotics available for clinical use has been rising as a principal public health problem around the world. In turn, this has evoked the reconsideration of bacteriophage therapy as an expedient way out of such predicament (Suh et al., 2022). Lytic phages, besides their noticeable antibacterial efficacy, are advantageous in multiple aspects as compared to lots of antibiotics. For example, they strictly reproduce at the infection site where their target host is most abundant, also they are distinguished with infrequent or even single administration requirement, sparing the patients lots of suffering, and definitely, the eminence of retaining activity against the otherwise pan-drug resistant bacteria. All these have contributed to the resurgence of interest in antibacterial phage therapy. Seeking an effective strategy to combat the multi-drug resistant organism, MRSA, using phage CCASU-L10, isolated in our previous study from chicken rinse and its optimization was the aim of this study (Berryhill et al., 2021; Gordillo Altamirano and Barr, 2021). Staphylococcal phages are usually obtained from sewage (Han et al., 2013; Li and Zhang, 2014; Abatángelo et al., 2017; Melo et al., 2018), and milk (García et al., 2009; Kwiatek et al., 2012). Presumably, the isolation of *S. aureus* bacteriophages from chicken is not common (Duc et al., 2020).

The method originally described for providing direction on both, the purity of a particular phage lysate, and the titer of viral particles existing is the Plaque assay (Anderson et al., 2011). Optimally, it results in discrete clear zones: plaques, each of them representing an infectious center (Ács et al., 2020). If the result of this assay gives rise to a single plaque morphology, in terms of shape and size, this orients towards containing one pure phage type. Conversely, if it results in different plaque forms, this leans towards having more than one virus. Besides, a mathematical equation could work out the amount of the virus present in a lysate using the count of certain plaque type, in PFU/ml (Anderson et al., 2011; Hyman, 2019). Upon calculating the original titers of our lysate, it was fairly high (>10^8^ PFU/mL) and equitably reproducible. CCASU-L10 lysate resulted in only 1 plaque morphology inferring the existence of a single virus. In addition, plaque morphology could distinguish between the two phage types. For example, lytic (virulent) phages are commonly characterized by clear transparent plaques, whereas lysogenic (temperate) ones sometimes exhibit turbid, opaque ones (Jurczak-Kurek et al., 2016; Park et al., 2020). Furthermore, few phages are marked with halos surrounding the plaques; usually interpreted by the production of certain enzymes that diffuse around the infectious center. These enzymes probably show activity against the bacterial cell wall and the biofilm formed by many (Dakheel et al., 2019; Vukotic et al., 2020). Inspecting CCASU-L10 plaques, they appeared small (2 to 5 mm), circular with entire margin, and completely clear, emphasizing the lytic activity CCASU-L10 possesses (Duc et al., 2020). However, it showed no halos, negating an anti-biofilm activity. Nonetheless this demands more confirmation by testing on biofilm-forming microorganisms (Dakheel et al., 2019; Vukotic et al., 2020).

In order for a phage to be qualified for therapeutic purpose, it usually must have some criteria; such as being strictly lytic, showing a reasonably broad host range, and, of course, good stability (Klumpp and Loessner, 2013). Accordingly, the next stage of this study was examining some of the main properties of CCASU-L10.

The bacteriophage host range could be defined by the genus, species and strains of bacteria it is able to infect and lyse (Hyman, 2019). This, clearly, is essential when using a phage therapeutically, and especially to treat critical infections. A phage specific to certain bacterial species is favored because it prevents disturbing organisms except the pathogenic one, leaving the host’s microbiome unruffled, hence sparing the patients lots of side effects. Therefore, talking about species, a phage with a narrow host range is preferable. But, within this species, a phage capable of lysing most strains, is, naturally, advantageous. This supports its appropriateness for empirical therapy, similar to broad-spectrum antibiotics (Hyman, 2019). The way a phage interacts with the receptors of a certain host determines its range of activity (Ross et al., 2016; de Jonge et al., 2019). Also, the typical way a phage is isolated normally appoints one host on which phages are grown, this probably contributes to their naturally limited host range (Hyman, 2019). In the current study, CCASU-L10 exhibited a polyvalent lytic activity, being effective against all the 22 MRSA isolates, hence it was chosen to complete the study.

The phage particles morphology serves as a guidance on their classification. Although numerous morphologies of bacteriophages have been reported, and notwithstanding the late propositions against the customary stipulation of having only one phage order (Turner et al., 2021), hitherto, *Caudovirales* is the order including tailed phages. The most common morphotypes are *Myoviridae, Siphoviridae* and *Podoviridae*. These are identified by double-stranded (ds) DNA and an icosahedral capsid, but are discerned through their tails. *Podoviridae: Podo*, a Greek word, meaning “foot”, refers to the short tail the phages of this family possess, about 20 × 8 nm. The tail is non-contractile, meaning that the viral genome is released into the host cytoplasm merely by ejection after lysosymes have created an opening in the cell wall and without contraction of the tail sheath (Podoviridae ~ ViralZone, 2011; Podoviridae, 2012; Alič et al., 2017). Examination of the negatively-stained phage specimens using TEM is the procedure used for phage particles visualization (Ackermann, 2012a). The concentrated and purified CCASU-L10 phage suspension was subjected for TEM (Clokie and Kropinski, 2009; Kalatzis et al., 2016). The morphological characteristics appeared as follows: it was tailed, so, it could be classified under the order *Caudovirales.* It had an icosahedral head and lacked both the head-to-tail connectors and the base plates, excluding the probability of being a member of *Myoviridae* (Han et al., 2013; Alič et al., 2017). It had a comparatively large head (73 nm), and was marked with a short tail (16 nm). Matching these features to those described on Viral Zone website (Podoviridae ~ ViralZone, 2011; Podoviridae, 2012) and according to the (ICTV)-ninth report guidelines (King et al., 2011), it was suggested that phage CCASU-L10 could be a member of the family *Podoviridae*. That said, the definitive technique when confirming phage type and classification is genome sequencing. Although the majority of *S. aureus* phages found in phage cocktails are known to belong to *Myoviridae* possibly due to their strong lytic effects (Kornienko et al., 2020), the majority of the *S. aureus-*specific phages described in literature are members of *Siphoviridae* family, and a smaller number belong to *Podoviridae* (Deghorain and Van Melderen, 2012; Klumpp and Loessner, 2013; Duc et al., 2020). Nevertheless, podoviral phages were reported to be the most preferable when it comes to therapeutic application (Duc et al., 2020). Two reasons are behind this claim: First, some of the *Siphoviridae* viruses can sometimes turn to the lysogenic life cycle, carrying the risk of transfer of virulence and resistance genes among bacterial strains. Second, though both are strictly virulent, *Podoviruses* have comparatively smaller genome than *Myoviridae* phages, that encodes for well-defined genes; however, *Myoviridae’s* large genome harbors genes that are still of unknown function (Deghorain and Van Melderen, 2012; Cui et al., 2017; Duc et al., 2020). Taken together, the proposed grouping of the obtained phage matched earlier studies that isolated *S. aureus Podo-viruses* (Alič et al., 2017; Zhang et al., 2017; Cha et al., 2019; Kornienko et al., 2020).

CCASU-L10 was checked for longevity. The lytic effect was sustained for 90 days at different temperatures. The physicochemical stability of phages is crucial when it comes to real-life application, large scale production, formulation, and storage (Abdelsattar et al., 2019; Abdallah et al., 2021). Ackermann explained that the behavior of tailed phages is less erratic under harsh conditions compared to other phage forms: the filamentous and the cubic (Ackermann et al., 2004). Since phage adsorption to the host cell, and its whole life cycle is impacted by factors, like pH, temperature, some chemicals, and irradiation (Mahmoud et al., 2020; Abdallah et al., 2021), CCASU-L10 was tested after challenge under some extreme physicochemical conditions. It showed overall satisfactory stability that can set it out for use at larger scales. It enjoyed good thermal survivability; thermal inactivation point was 60°C after 1 hr exposure, which may facilitate the shelf storage and increase the tolerability to hot weather. This coincided with the stability examination performed by González et al. where they reported the loss of infectivity of their phages after 90 minutes exposure to 60°C (González-Menéndez et al., 2018b). It also resembled the stability of another *Podo*virus active against *Yersinia*, that was inactivated at 50-minute exposure to 60°C (Filik et al., 2020). This level of thermal stability outstands that of some popular *S. aureus* phages studied previously such as MSP (Ganaie et al., 2018), SPW (Li and Zhang, 2014), and SAJK-IND (Ganaie et al., 2018), which got completely deactivated above 50°C. Nevertheless, some other investigations stated higher thermal inactivation points; for example, 65 °C was reported by Wang et al. (Wang et al., 2016). In addition, a phage called vB_SauM_CP9 retained infectivity till 70 °C (Abdallah et al., 2021). Mahmoud et al. observed extraordinary thermal stability up to 85 °C when studying their *S. aureus* phages (Mahmoud et al., 2020).

The phage CCASU-L10 showed a fluctuating activity along the pH spectrum. The clear spot vanished at the acidic extreme (pH:1 and 2). It retained the lytic activity along the pH range: 3-11. However, it fell off again at the alkaline end of the spectrum (pH: 12 and 13). This was like the pattern stated in the research performed on an anti-*Yersinia podo*virus in 2018 (Filik et al., 2020). In the same study on the bacteriophage vB_SauM_CP9, it could not survive outside the pH range: 4-9 (Abdallah et al., 2021). Compared to the former and to another staphylococcal phage recovered by Wan et al., which survived only over a narrow range (pH: 6-8), CCASU-L10 showed way greater stability (Wan Nurhafizah et al., 2017). On grounds of this, it can be concluded that CCASU-L10 has great stability over a broad pH range.

UV has been uprising as a method of disinfection, of water and of the surfaces as well (Rodriguez et al., 2014). Thus, it might be a part of the phage industry in the future. However, the UV light elicits genomic damage, mutations, and recombination. Therefore, UV was well-recognized to be one of the most harmful factors on phages in very short times (Kim et al., 2018). Accordingly, the UV irradiation effect on phage CCASU-L10 was evaluated. In fact, CCASU-L10 remained active up to 30 minutes of exposure to UV, the activity declined after this point in time till disappeared after 1 hr, supporting the mentioned theory that UV, definitely, imposes a depreciating harm on this phage. This observation came in agreement with other reports (Rodriguez et al., 2014; Kim et al., 2018; Hussein et al., 2019).

Some organic solvents are well-known for their viricidal effect. The effect of some common organic solvents (ethanol, isopropanol, and chloroform) was tested on the phage which survived all the concentrations examined, but got inactivated by the 100% isopropanol. This concurs with other reports (Kim et al., 2018).

In order to use phages therapeutically, there is a growing demand to have an access to systematic information on the optimal conditions that facilitate their mass production. Lytic phages are the principal type in therapeutic application. And since phage propagation relies on the bacterial host's physiological nature and its multiplication rate (Hadas et al., 1997; Kick et al., 2017; Nabergoj et al., 2018), the slightest variations in the key process variables like the culture conditions, and media influence their production (Egli, 2015; Nguyen et al., 2021). So, to ensure the reproducibility of the production process with a high phage yield, handling the phage-host interaction, and the co-culture could be a promising scaling-up strategy. Hence, optimization of phage CCASU-L10 production on laboratory scale was the last step in this study (Grieco et al., 2012; González-Menéndez et al., 2018b; Kim et al., 2020). However, practically deciding on the ideal parameter combination of a certain process is costly and time-consuming. Making use of the advent of biotechnology, along with the notion of the design of experiments (DOE) can facilitate the production optimization (Grieco et al., 2012). DOE is a tool used for optimizing the bioprocesses, it studies the variables that influence an outcome (Grieco et al., 2012). The classical OFAT method studies the factors influencing the production individually. However, it is expensive, tedious, and depends on plenty of experiments. Moreover, such conventional methods suffer compromised accuracy, cannot measure the interactions between multiple factors, and could mislead on the optimal settings. Thus, the OFAT approach, solely, is not dependable, if it is to completely capture the bacterium-phage system behavior (Latha et al., 2017). Moreover the development of modern optimization approaches, virtual and computational ones, have revolutionized the concept of experiment design; they have introduced vibrancy, and ensured robust accuracy. The advanced RSM, which is concerned with the combined effect of more than one factor, is therefore befitting when optimizing processes affected by a combination of parameters. In addition, it is now done with the help of design software, saving time and cost.

In light of the aforementioned, the present study has sought phage CCASU-L10 production optimization combining both OFAT and RSM, in order to identify the optimal settings necessary for enhancing its yield, and obtaining desirable results. Two different sources for each of the carbon and nitrogen were tested, since they are usually vital media components. Glycerol and peptone were favored by phage CCASU-L10. This agreed partially with a previous study on the staphylococcal *Kayvirus* psa-3, which stated that glycerol and glycine were the preferred sources (Kim et al., 2020).

Contrary to expectations, CCASU-L10 leaned towards the inoculum size of 10^7^ CFU/ml. This was close to the result announced by Kim et al. in the same research, who chose 10^8^ CFU/ml to be the optimum level (Kim et al., 2020). Temperature is a crucial culture condition. Accordingly, three levels of temperatures were examined. Surprisingly, 28 °C resulted in maximum phage titer. This was incongruous with another study which declared 38 °C to be the temperature conducive to the best production of their phage (González-Menéndez et al., 2018a). RSM was applied thereafter, and the input variables incorporated for examination were: concentration of glycerol, concentration of peptone, and pH. Each parameter was tested at three different levels and the D-optimal design was selected using the statistical software package Design Expert^®^ v. 7.0. This design settled on the optimal, yet doable number of experiments (González-Menéndez et al., 2018a). Taking on the experimental design suggested, 17 runs were carried out to study the influence of the above factors at the same time. A quadratic model was created.

Afterwards, ANOVA was performed to estimate the model significance and validity (Bhaumik et al., 2013). The obtained F-value was 18.76 (P-value <0.0002), proving the model significance. The coefficient of determination R^2^ is a statistical measure of fit; it denotes how much variation in response could be explained by a model. It ranges between zero and 1, and is normally conveyed in percentage (Chen et al., 2009; El-Sayed et al., 2020a); where 1, the maximum, shows that 100% of the variation could be interpreted by the model. The attained R^2^ (R^2^ = 0.94) substantiates that the generated model could explain more than 90% of the variability that may result in response. The adjusted R^2^ (Adj R^2^) is an analogue of R^2^ that is altered according to the number of predictors in a model. It was 0.8988. One more coefficient is the predicted R^2^ (Pred R^2^). It shows how well a model predicts responses for new data. It should be congruent with Adj R^2^ with a difference equals or less than 0.2 (El-Sayed et al., 2020b). This, too, applies in the generated model, as it was recorded to be 0.7766. Adequate precision is the signal to noise ratio, it supports proper discrimination by the model. A ration greater than 4 is preferrable (Abdel-Hafez et al., 2014). It had the value 14.536. Moreover, the model had a low coefficient of variance (C.V.%): 1.45, emphasizing the reliability of the model (El-Sayed et al., 2020a; El-Sayed et al., 2020b). The software plotting utilities generated model diagnostic plots and these, too, confirmed its validity. Each of the terms A and B, and the interactions: AB, AC, BC, and A^2^, all had P-values <0.05, thus they were discerned as significant parameters that impact phage production. This showed that only two factors (pH, and glycerol concentration) influenced the production enhancement target. Interaction is a situation where one factor effect relies on the level of another. All interactions between the factors affected the phage yield significantly. The optimal combination of variables essential for maximum response was estimated *via* navigating the 3D plot presentation of the model. This, along with the numerical optimization function offered by the program, commended the following optimum conditions as conducive to the highest phage titer. These conditions were pH 8, glycerol 0.9%v/v, and peptone 0.08%w/v. Indeed, when verified experimentally, these conditions successfully enhanced the output of the produced phage titer (1.17x10¹² PFU/ml) by two-log fold compared to the unoptimized conditions. Fundamentally, the optimization process can be regarded as a potential scale-up strategy.

In light of the revived interest in phage therapy as a substantial supplement to the antibacterial ammunition, it is crucial to point out that the phage-bacteria co-evolution must be considered while evaluating the application of the phage therapy (Laanto et al., 2017; Ofir et al., 2018; Rohde et al., 2018). Recently, two bacterial phage resistance mechanisms, BREX (BacteRiophage Exclusion) and DISARM (Defence Island System Associated with Restriction-Modification), were discovered within some bacterial genomes at variable ratios. That said, what still makes phage a unique antibacterial agent, in contrast to the static chemical compounds, is that it can dynamically adapt to the newly developed resistance systems in bacteria and evade it relying on various strategies such as anti-CRISPR (Clustered Regularly Interspaced Short Palindromic Repeats) proteins. Moreover, such resistance could be diminished by using cocktails of phages characterized by broad host range, high adsorption rates, and large burst sizes. Another method that can decrease the risk of resistance towards phages is paying more attention to the phage effective endolysins (Schmelcher et al., 2012; Rohde et al., 2018).

## 5 Conclusion

The scenario of antimicrobial resistance has become dreary over the past decades. The world now is in a crossroads where it has to contrive a coherent course of actions, if we are to survive through such hard time without much loss. This has prompted both the scientific and the clinical communities to seek novel and efficient alternatives to antibiotics such as bacteriophage. In this study, a bacteriophage isolated from raw chicken rinse succeeded in lysing 23 clinical MRSA isolates originating from various specimens. This phage is suggested to be classified under the chief order *Caudovirales*, family *Podoviridae*, and displayed a reasonable stability facing some extreme conditions like high temperature, extremely acidic and alkaline media, and direct UV irradiation, probably qualifying it for future *in-vivo* efficacy check. Moreover, phage yield optimization using OFAT and RSM resulted in a considerable increase in the titer produced as compared to the regular culture conditions. Altogether, phages, definitely, are one of the nature’s gifts encountering the AMR threat. It is on us to make good use of such a promising recourse by increasing concern, further exploration and conducting reliably-designed clinical trials.

## Data availability statement

The raw data supporting the conclusions of this article will be made available by the authors, without undue reservation.

## Author contributions

Conceived and designed the experiments: I-AA, GSH, KMA. Carried out the experiments: I-AA. Carried out the statistical analysis, GSH. Drafted the paper: I-AA, GSH, KMA. Wrote the paper in its final format: GSH, I-AA, KMA, NAH, MAA, HR. All authors contributed to the article and approved the submitted version.
